# Effect of Printing Temperature on the Microstructure and Tensile Properties of Polylactic Acid–Magnetic Iron Composites Manufactured by Material Extrusion

**DOI:** 10.3390/polym17182485

**Published:** 2025-09-14

**Authors:** Meriem Bouchetara, Sofiane Belhabib, Alessia Melelli, Jonathan Perrin, Timm Weitkamp, Ahmed Koubaa, Mahfoud Tahlaiti, Mustapha Nouri, Sofiane Guessasma

**Affiliations:** 1 INRAE, Research Unit BIA UR1268, Rue Geraudiere, F-44316 Nantes, France; meriem.bouchetara@inrae.fr; 2 UQAT, IRF, Campus de Rouyn-Noranda, Rouyn-Noranda, QC J9X 5E4, Canada; ahmed.koubaa@uqat.ca; 3 Nantes Université, CNRS, GEPEA, UMR 6144, F-44000 Nantes, France; sofiane.belhabib@univ-nantes.fr; 4 Synchrotron SOLEIL, F-91190 Saint-Aubin, France; alessia.melelli@synchrotron-soleil.fr (A.M.); jonathan.perrin@synchrotron-soleil.fr (J.P.); timm.weitkamp@synchrotron-soleil.fr (T.W.); 5 Icam School of Engineering, Nantes Campus, 35 Av. du Champ de Manœuvres, F-44470 Carquefou, France; mahfoud.tahlaiti@icam.fr (M.T.); mustapha.nouri@icam.fr (M.N.)

**Keywords:** additive manufacturing, PLA-magnetic iron, microstructure, X-ray microtomography, tensile performance, finite element computation, printing temperature, magnetic actuation

## Abstract

In this study, we examined how printing temperature affects the microstructure and mechanical properties of polylactic acid (PLA) composite reinforced with iron oxide i.e., magnetite manufactured using a material extrusion technique. The composite was printed at temperatures from 185 °C to 215 °C. Microstructure analysis via synchrotron radiation X-ray microtomography revealed changes in both iron oxide and porosity contents within the printed structures. Mechanical testing results demonstrated a limited effect of the printing temperature on tensile performance. Finite element computation is considered to predict the elasticity behavior of the printed composite by converting 3D images into 3D structural meshes. When implementing a two-phase model, the predictions show a leading role of the iron oxide content, and an overestimation of the stiffness of the composite. A three-phase model demonstrates a better matching of the experimental results suggesting a limited load transfer across the PLA-iron oxide interface with Young’s moduli in the interphase zone as small as 10% of PLA Young’s modulus. Magnetic actuation demonstrates that experiments on PLA-iron oxide plates reveal a pronounced thickness-dependent limitation, with the maximum deflection observed in thin strips of 0.4 mm.

## 1. Introduction

Additive Manufacturing (AM) has emerged as a new technology for material architecting, attracting significant attention in recent years [1,2]. It offers substantial potential for producing highly intricate technical components with precision, layer by layer, from digital models [3]. AM enables localized material deposition, allowing for customized parts with minimal dependence on traditional tooling [4,5,6]. This has paved the way for the creation of innovative materials, including adaptive materials [7]. With its swift fabrication cycle, AM is applied across a wide range of industries, including bioengineering, aeronautics, civil engineering, prototyping, automotive, the food industry, and art [8,9,10,11,12]. The widespread adoption of AM is due, in part, to its versatile processes, which allow for the printing of a diverse range of materials. Among these, material extrusion is particularly notable as a popular and cost-effective method for printing polymer structures [13,14].

At the beginning of material extrusion, PLA (Polylactic acid) and ABS (Acrylonitrile Butadiene Styrene) have become key filament materials in additive manufacturing [15,16]. Research, such as that by Ahn et al. [17], has highlighted the significance of part orientation in inducing anisotropic behavior in ABS-printed materials. Advances in material extrusion or commonly named fused filament fabrication (FFF) have increasingly focused on high-performance feedstock materials [18], including ceramic-based composites and carbon fiber-reinforced composites [19,20,21,22], to mitigate the typical reduction in mechanical performance caused by process-related porosity. These studies demonstrate that the benefits of incorporating a secondary material can be diminished if optimal printing conditions are not maintained.

Metal-reinforced polymers have also attracted considerable interest [21]. For instance, Buj-Corral et al. [23] examined the effects of porosity in copper-reinforced PLA, while Martinez et al. [24] explored the development of iron-filled PLA for microwave absorption applications using 3D printing technology. Török et al. [25] investigated how FFF parameters influence the mechanical performance of various metal-reinforced PLA composites, including those with copper, steel, and iron fillers. Both Buchanan et al. and Kumar et al. [26] studied how process conditions, such as infill density, layer height, and print speed, affect the properties of iron-PLA composites. Their research concluded that optimizing the printing process, such as by reducing infill density, increasing layer height, and increasing print speed, could improve print efficiency. Among the other feedstock materials that received great attention is magnetic polymer composites. Indeed, the growing relevance of magnetic polymer composites is highlighted in various fields, from engineering to medicine [27]. These hybrid materials combine the properties of polymers and magnetic substances, providing a unique array of functionalities and innovative applications, such as additive manufacturing, 3D/4D printing, and soft actuators in robotics that can be activated by external stimuli [27,28,29].

This research explores the potential of material extrusion in developing adaptive composite materials capable of magnetic actuation. Adaptive composites are materials that can alter their properties in response to external stimuli, such as shape memory alloys and hygromorphs [30,31]. For magnetic actuation to be efficient, it must be performed under low mechanical stress due to the weak magnetic fields generated [32].

Modulating mechanical performance is crucial for magnetic actuation but is constrained by solid-phase connectivity, mechanical stability, and service requirements. Among the various printing parameters, part orientation is key, as it influences load transfer. As Sood et al. [33] have shown, load transfer is significantly affected by the building direction, necessitating filament arrangements perpendicular to this direction. Printing temperature also influences stretchability and strength, though this effect may be limited, especially for composite filaments [34,35]. The infill rate is another critical parameter, but the effectiveness of deformation mechanisms depends largely on the type of unit cell used for the infill [36].

The need to understand spatial heterogeneity and examine the internal structure of magnetic polymer composite materials at different resolution scales is attracting significant interest to optimize their architecture and improve their functionalities [37,38]. Indeed, an exhaustive literature analysis (Supplementary Material) using the Web of Science database within the field of additive manufacturing shows that only seven documents (less than 1% of the total literature on the subject) addressed addressing magnetic materials.

X-ray microtomography (XMT) is a non-destructive imaging technique used to explore the internal structure of materials [39,40,41]. Synchrotron radiation-based 3D imaging offers unparalleled resolution and depth, allowing precise visualization of intricate microstructures, including second-phase distribution and porosity [39,42]. XMT not only helps in understanding the relationship between microstructure and mechanical properties but also assists in optimizing printing parameters for improved material performance. In this context, while the mechanical performance of metal-reinforced feedstock materials has been widely reported, there is a noticeable lack of studies that establish a clear correlation between tensile behavior and microstructural features such as porosity and interlayer bonding quality. Such correlations are essential for understanding how processing conditions at the microscale affect the resulting macroscopic properties.

In this context, the primary objective of this study is to analyze the microstructure of 3D-printed PLA-iron oxide composites in relation to printing temperature and its implication in tensile performance. From a manufacturing perspective, the printing temperature is a readily adjustable parameter. Understanding its isolated impact on metal-filled composites supports optimization strategies for functional prototyping and end-use parts, which remains highly relevant for practitioners and engineers. Finite element computation is considered to explain the observed behavior and identify a mechanical model that explains the link between the microstructure and the elasticity behavior.

## 2. Experimental Layout

### 2.1. Materials and Processes

The material used in this study is a 1.75 mm magnetic filament from Proto-Pasta (Vancouver, WA, USA), composed of polylactic acid (PLA) modified with magnetic iron fillers (Rustable Magnetic Iron). The magnetic characterization of the as-received material indicates a saturation induction of approximately 0.15 T. The relative permeability, compared to vacuum (µ = 4 × π × 10^−7^ H/m), ranges between 5 and 8 and remains consistent across frequencies up to 1 MHz. Similarly, the absolute magnetic permeability lies between 62 × 10^−7^ and 100 × 10^−7^ H/m, showing no significant frequency dependence within the same range.

Two types of magnetic PLA-iron oxide composite specimens were fabricated using material extrusion (Ender-3 V3 printer from Shenzhen Creality 3D Technology Co, Ltd., Shenzhen, China) [32,43]: dog-bone specimens for tensile testing, and rectangular specimens for magnetic actuation experiments. Each sample was pre-modelled using the computer-aided design (CAD) software SolidWorks v2022, and converted into surface tessellation (STL) format. The printed tensile specimens had dimensions of 80 × 20 × 4 mm, which conform to ISO 527-1/-2 standards [44]. The rectangular strips had a similar cross-section of 83 × 22 mm^2^ and a varied thickness between 0.4 and 0.8 mm. The specimens were printed with a specific orientation (layups −45°/+45°) to improve the final tensile properties [45,46,47]. The other printing conditions are as follows: bed temperature set to 60 °C, printing speed set to 30 mm/s, nozzle diameter 0.4 mm, layer height of 0.2 mm, and the printing orientation is horizontal. The main printing condition studied is the printing temperature. This study specifically focuses on the effect of printing temperature over a broad range as this is a critical parameter influencing both material printability and process efficiency. Moreover, printing temperature plays a key role in optimizing energy consumption during additive manufacturing. Within this temperature range, the thermal behavior of PLA reinforced with magnetite particles is expected to show significant variation due to the stark contrast in thermal properties between the polymer matrix and the metallic reinforcement.

In this study, this parameter is increased from 185 °C to 215 °C with a step of 10 °C. Below 185 °C, the printability limit of PLA is met, and above 215 °C, overheating also causes the filament to become overly fluid, leading to poor dimensional accuracy of the printed dog-bone part. Before the printing process, the commercial filament was conditioned in a desiccator to ensure uniform preparation.

### 2.2. Tensile Testing

**Figure 1:**
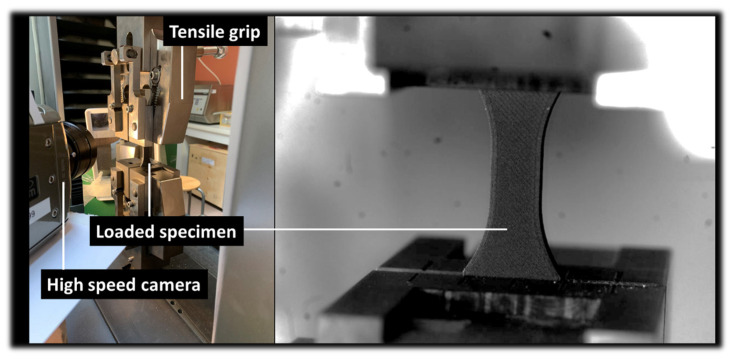
Sample geometry and tensile testing of 3D-printed PLA-iron filament.

The mechanical properties of the PLA-iron composite were determined using a Zwick/Roell universal tension/compression testing machine (Zwick-Roell Group, Ulm, Germany), equipped with a 10 kN load cell. The displacement speed was set at 5 mm/min, and sample deformation was recorded (Figure 1).

Sixteen samples were tested with four repetitions for each 3D printing temperature condition. Tests were carried out at room temperature (25 ± 5 °C) and relative humidity ranging from 55% to 65%. Deformation sequences are observed using the optical high-speed camera Phantom V7.3 from Photonline company (Pacé, France). The entire loading sequence is recorded in full frame (800 × 600 pixels) at a moderate speed of 100 fps (frames per second).

### 2.3. Scanning Electron Microscope (SEM)

SEM analysis is conducted to obtain a quantitative assessment of the 3D-printed structures, with a primary focus on identifying and evaluating the presence of defects.

SEM analysis is performed on 3D-printed samples to examine the influence of printing temperature on the crack patterns resulting from tensile loading. The analysis is carried out using a JEOL JSM-5800LV microscope (JEOL Ltd, Akishima, Japan) with an accelerating voltage of 15 kV. To ensure surface conductivity during observation, the samples are coated with a 50 nm carbon layer using a Balzers CED 30 carbon evaporator (OC Oerlikon, Balzers, Liechtenstein). The magnification is set between 35× and 300×, with typical pixel sizes between 0.37 µm and 3.13 µm.

### 2.4. X-Ray Microtomography Imaging

The objective of the X-ray microtomography analysis is to provide deeper insight into the 3D structure of the printed composite, with a focus on quantitatively evaluating defect content and reinforcement distribution, and how these features are influenced by printing temperature. X-ray microtomography analysis of the magnetic composites was conducted using synchrotron radiation-based X-ray microtomography at the ANATOMIX beamline (Synchrotron SOLEIL, Saint-Aubin, France). This analysis has two main targets, (i) investigate the microstructural factors influencing the mechanical performance of magnetite-filled PLA composites; (ii) combine high-resolution synchrotron X-ray microtomography with finite element modelling to predict how printing temperature affects internal structure and mechanical behavior.

The samples were mounted onto magnetic support holders using simple fixtures. Inline phase contrast (propagation distance 1 m) was employed to perform microtomography. A filtered white beam (transmission filter 1 mm Cu, undulator gap 7 mm) with a central energy around 50 keV was used. The detector was based on an indirect scheme with a scintillator (lutetium aluminum garnet, 600 µm thick) coupled via lens optics (two Hasselblad photo objectives in tandem geometry 1:1) to a CMOS ORCA Flash 4.0 V2 camera (Hamamatsu, Japan) operated in full-frame unbinned mode (2048 × 2048 pixels). The resulting effective pixel size was 6.5 µm. The exposure time was set to 50 ms per projection radiograph. A standard 180° scan geometry was used with 2000 projection angles per scan. Reconstruction was performed using PyHST2 software (version 2019b), with Paganin correction applied (Paganin length: 100 pixels, unsharp filter length: 3 pixels, unsharp filter coefficient: 0.7). The full reconstructed volumes with an initial size of 2048 × 2048 × 2048 voxels were then cropped to a region of interest capturing the full width (10 mm) and thickness (4 mm) of the specimen, along with a representative length of approximately 9 mm. This resulted in a final size of the volume of interest of around 1.75 × 10^9^ voxels per acquisition.

**Figure 2:**
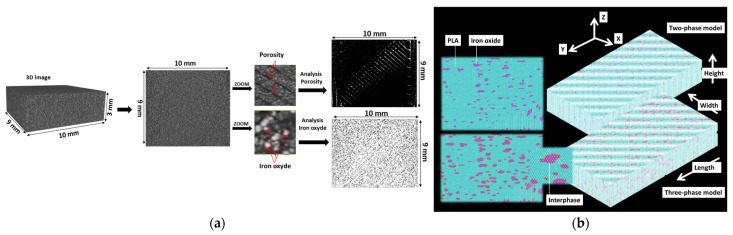
Main image processing and finite element computation processes, (**a**) summary of main image processing associated with X-ray microtomography imaging of 3D-printed PLA-iron oxide composites; (**b**) finite element meshes corresponding to two-phase and three-phase models.

The resulting 3D tomographic images were extensively analyzed with ImageJ software version 1.53 (NIH, Bethesda, MD, USA) [48], using internally developed macros. The study aimed to investigate the distribution of porosity and iron particles within the PLA matrix. Parameters such as spatial distribution, phase content (iron oxide and porosity), connectivity, dispersion, and morphology were quantified (Figure 2a).

Segmentation is performed based on a double threshold process to isolate both iron particles and the porosity from the PLA matrix and the background. The result of the segmentation is a binary image where the grey level $\gamma_{\phi (ijk)}$ distribution for each phase ϕ corresponds to either the background value or the phase value. Based on the segmentation result, the apparent volume content is calculated as a voxel count. Indeed, the phase content accuracy is limited by the spatial resolution of the synchrotron radiation experiment. Since the voxel size used is 6.5 µm, any features smaller than this value cannot be detected or quantified.

The apparent phase content is measured as sum of the voxels belonging to each phase according to the formula.

For porosity in white color (ϕ=P):

(1a) $$f_{P}\left(\%\right)=\sum_{i,j,k=1}^{D_{X}\times D_{Y}\times D_{Z}}\left(\gamma_{P(ijk)}/255\right)/\left(D_{X}\times D_{Y}\times D_{Z}\right)$$

For iron oxide in black color (ϕ=I):

(1b) $$f_{P}\left(\%\right)=\sum_{i,j,k=1}^{D_{X}\times D_{Y}\times D_{Z}}\left(1-\left(\gamma_{P(ijk)}/255\right)\right)/\left(D_{X}\times D_{Y}\times D_{Z}\right)$$

where $f_{\phi }$ represents the phase content in the domain bounded by ($D_{X}$,$D_{Y}$,$D_{Z}$), i.e., total number of voxels in the X-, Y-, and Z-directions. $\gamma_{\phi ijk}$ denotes the grey level associated with the voxel at coordinates i, j, and k for phase ϕ.

As indicated in Equations (1a) and (1b), the determination of phase content is performed through voxel counting. Consequently, the obtained values represent the apparent volume fraction of both the reinforcing phase and the porosity within the resolution constraints of the imaging method. Therefore, the measured volume fraction does not capture contributions from sub-voxel features.

The axial porosity level is measured as an estimation of the anisotropy of phase content. The relative change in one of the three main directions X, Y, or Z is quantified as follows. For instance, in X-direction this writes,

(2) $$f_{\phi X}\left(i\right)=\sum_{j,k=1}^{D_{Y}\times D_{Z}}\left(1-\left(\gamma_{\phi (ijk)}/255\right)\right)/\left(D_{Y}\times D_{Z}\right)$$

where $f_{\phi X}\left(i\right)$ is the porosity level in the ith position along the X-direction.

Segmented images are further processed to derive the homogeneity of phases. Grey level images are built where each voxel from the background encodes the distance to the closest feature. The result is a planar Euclidian distance map for both the porosity and iron oxides according to the equation,

(3) $${\gamma^{\prime }}_{\phi (ijk)}=255\times \left(Max\left(d_{ijk}\right)-d_{min}\right)/\left(d_{max}-d_{min}\right)$$

where ${\gamma^{\prime }}_{\phi (ijk)}$ is the new grey level associated with voxel (ijk) from phase ϕ, $d_{min}$, $d_{max}$ are the minimum and maximum distance from the features of interest, and $d_{ijk}$ is the distance encoded for voxel (ijk).

From the cumulative histogram of the new grey levels, the slope is derived as a measure of the homogeneity of phase distributions. A flat curve indicates clustering while a steep one would mean a well-dispersed phase.

The connectivity of the phases is assessed through 3D labelling of all phases. This approach enables the identification of the largest connected feature and allows for the calculation of the connectivity index, which is defined as the ratio of the largest feature volume to the total volume of features for a given phase. The connectivity index is expressed as follows:

(4) $$C_{\phi }\left(\%\right)=100\times V_{max}/\sum_{n=1}^{N}V_{n}$$

where $C_{\phi }$ is the connectivity index for phase ϕ, $V_{max}$ is the volume of the largest feature, $V_{n}$ is the volume associated with labelled feature n among the identified population, and N is the number of labelled features.

## 3. Finite Element Model

Finite element computation is considered able to gain more knowledge about the correlation between the microstructure induced by 3D printing of PLA-iron oxide composites and the tensile performance. Finite element simulation is designed to test different hypotheses about the role of the phase arrangement and the interface region in the printed composite, particularly how it influences the apparent stiffness as a function of iron oxide distribution and porosity.

From X-ray microtomography, 3D images are converted into structural meshes using the voxel-to-element conversion scheme. The solid phase is meshed uniformly, with structural cubic elements defined by eight nodes, each with three displacement components (UX, UY, and UZ) per node. Various sample sizes and resolutions are analyzed to assess the influence of microstructural details and the representative elementary volume. The effect of resolution was examined for parallelepipedic volumes between 150 mm^3^ and 257 mm^3^. The resolution reduction, defined as the ratio of final to initial resolutions, ranged from 0.05 to 0.32, with voxel sizes varying from 20 to 130 µm. Additionally, the representative elementary volume is investigated by cropping progressively larger volumes within the samples. A sampling ratio, defined as the ratio of the cropped volume to the total volume, is varied from 0.1 to 0.5, corresponding to volumes ranging from 0.4 mm^3^ to 6.1 mm^3^. The model size varies from 0.56 million to 86 million degrees of freedom (dofs), depending on the resolution and sample volume.

An isotropic elastic material model is used to evaluate the tensile behavior of the 3D-printed PLA-iron oxide composite. Young’s modulus and Poisson’s coefficient of both PLA matrix (1.092 GPa, 0.33) and iron oxide (215 GPa, 0.26) are implemented in the model based on experimental data and the technical sheet from the supplier. These correspond to the case of a two-phase model. Additionally, a second model is introduced to account for an imperfect interface between the matrix and the reinforcement (Figure 2b). This model assumes that the matrix region adjacent to the reinforcement has reduced stiffness. As a result, an additional material model is incorporated into a three-phase model, where Young’s modulus near the reinforcement varies significantly, typically between 0.1 GPa and 284 GPa. In the model, a fixed interphase thickness corresponding to a voxel size of 32.5 µm is used. The optimal interphase conditions are determined by varying the interphase modulus to achieve the best agreement between the experimental and numerical composite moduli. The chosen thickness represents the minimum element size used in the simulation, although a larger interphase thickness could also be considered by appropriately adjusting the interphase modulus. As highlighted in previous studies by the authors [49,50], the contribution of the interphase to the composite stiffness is governed by the ratio E_I_/t_I_, where E_I_ is the interphase modulus and t_I_ is the interphase thickness.

The computation is based on a quasi-static linear model that predicts the stiffness of the 3D-printed PLA-iron oxide composite stiffness in relation to a varying content of porosity and iron oxide. Tension is simulated along the main directions of the 3D-printed composite. For a typical loading along the length of the specimen, i.e., X-direction, the following lateral displacements restrictions are implemented (UY = 0 and UZ = 0) at both ends (x = 0, x = L, where L is the sample length), applying a positive displacement (UX = U > 0) at nodes for x = L, and fixing UX = 0 at nodes at x = 0. The elasticity problem is solved using a Preconditioned Conjugate Gradient (PCG) solver, with the overall Young’s modulus determined from the reaction force at the loaded end (x = L). Stress fields are also derived to assess how the microstructure influences stress distribution. Due to the large scale of the models, high-performance computing resources with CPUs running at 5.2 GHz and 128 GB of RAM are used, with computation times reaching up to 80 min per load increment. Ansys multi-physics software (version 2022 R2) is used for all simulations. A typical simulation run lasts around 1 h for loadings considered along the length of the sample (X direction).

## 4. Magnetic Actuation Experiment

**Figure 3:**
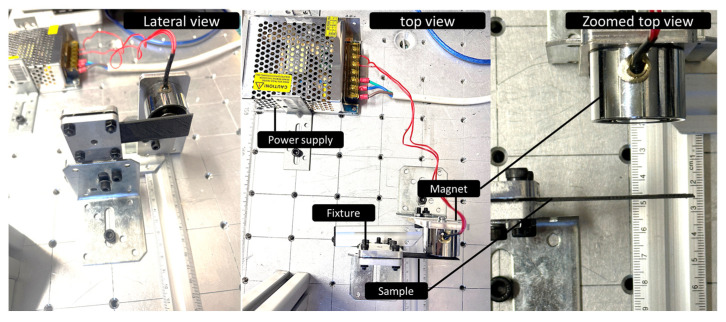
Setup of the magnetic actuation experiment showing the electromagnet and the 3D-printed strip used for displacement measurements.

A magnetic actuation experiment was performed to evaluate the displacement induced by an applied magnetic field. Since the printing temperature showed only a minor influence on the material stiffness, only samples printed at 200 °C were considered. Figure 3 illustrates the experimental setup. An electromagnet with a cylindrical shape, a load capacity of 150 N, and a diameter of 20 mm was positioned in front of a 3D-printed strip measuring 83 mm × 22 mm, with a thickness ranging from 0.4 to 0.8 mm. A switch controlled the current supply to the electromagnet, which was powered at 12 V. The lateral displacement of the strip was recorded as a function of the relative distance between the electromagnet and the 3D-printed sample.

## 5. Results and Discussion

### 5.1. Mechanical Behaviour of 3D-Printed PLA-Iron Oxide Composites

**Figure 4:**
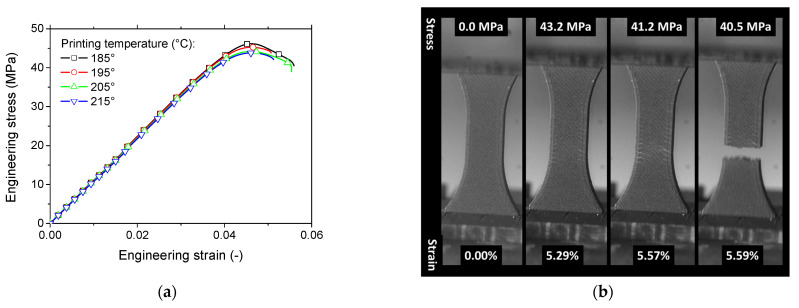
Tensile response of 3D-printed PLA-iron oxide samples, (**a**) engineering stress versus engineering strain as a function of the printing temperature; (**b**) typical deformation sequence captured using optical recording.

Figure 4a presents the engineering stress–strain response for 3D-printed PLA-iron oxide under tensile loading, showing the effect of different printing temperatures. All tested conditions exhibit elastic behavior followed by a modest plastic phase.

The elastic-like response can be attributed to the porosity induced during the manufacturing process and the influence of the +45°/−45° printing orientation. Hanon et al. [51] reported similar behavior in PLA tensile specimens printed with this orientation, characterized by a distinct yield limit without a subsequent hardening phase. Comparable findings were also noted by Hsueh et al. [52]. Furthermore, the printing temperature appears to have a minimal effect on the material’s ductility.

Among the tested samples, the composite printed at 185 °C shows a slight improvement in tensile strength compared to others. However, the elasticity stage analysis reveals that the stress–strain curves are nearly indistinguishable, indicating that the printing temperature does not significantly affect the material’s elasticity. These trends are further supported by the deformation sequences captured through optical imaging (Figure 4b). The variation between the ultimate stress at rupture and maximum stress is attributed to the diffuse damage pattern emerging from the specimen’s external frame (Figure 4b). At the same strain level corresponding to maximum stress, light grey regions, indicating microcrack formation, appear in the deformed specimens. These microcracks propagate perpendicularly to the loading direction at a moderate speed, as shown in Figure 4b.

A quantitative summary of the printing temperature’s impact is provided in Table 1, detailing key engineering parameters such as Young’s modulus, yield stress, tensile strength, ultimate stress, and elongation at break).

The stiffness test results show a consistent trend across all printing temperatures, with variations below 1%, indicating that stiffness is not influenced by printing temperature. Similar trends are observed for other parameters like ultimate properties, mechanical strength, and yield stress, with a sensitivity of less than 2% across all conditions. However, as shown by Delbart et al. [53], in 3D-printed PLA reinforced with carbon black, the degree of crystallinity can increase by up to 15%, influenced by process parameters such as nozzle diameter and layer height. This suggests that while printing temperature alone may not markedly affect the mechanical response in our case, other processing variables can strongly influence PLA crystallinity and, consequently, its mechanical performance.

### 5.2. Fractography Analysis Using SEM

**Figure 5:**
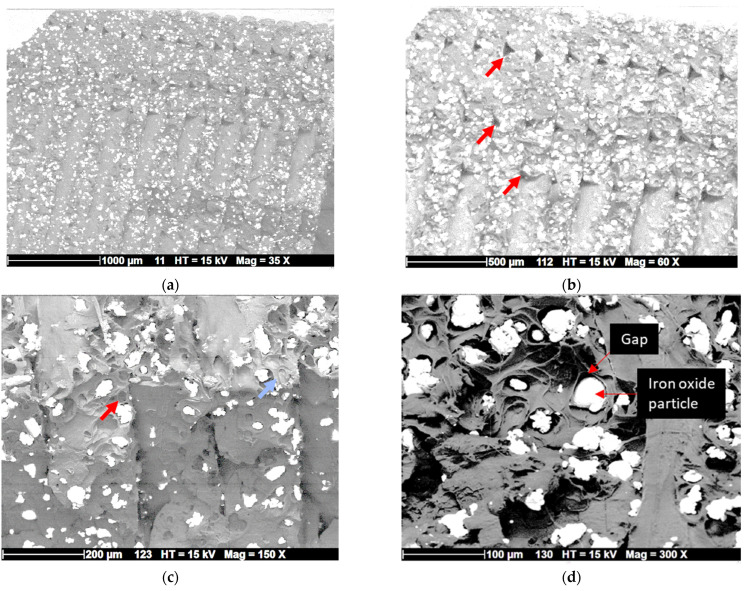
SEM micrographs showing the microstructure of 3D-printed PLA-iron oxide composites at different magnifications, (**a**) overall top view at low magnification showing both external frame and sample core, red arrows indicate gaps within filaments; (**b**) a magnified view on the filament arrangement; (**c**) lateral view showing the cracking pattern where the red arrow highlights particle pull-out and the blue one refers to filament tearing; (**d**) a close view on iron oxide particle debonding.

Figure 5 presents the microstructure of 3D-printed PLA composites with iron oxides, as shown by SEM micrographs, which highlight the dispersion of iron oxide particles within the PLA matrix. A uniform distribution of micrometric iron oxide particles is observed within the filaments (Figure 5a), appearing as a white-grey phase due to their high electrical conductivity. Although particle size estimation is not ideal without flat surfaces, the average particle size is roughly a few microns, with an estimated size of around 15 µm.

In addition to the iron oxide phase, the SEM micrographs reveal typical gaps between filaments, particularly at the junction between the external frame and the core of the specimen—defects that are common in fused filament fabrication (Figure 5b). However, the extent of these defects appears to be limited. Similar results were observed across different printing temperatures, indicating the limited influence of printing temperature on the microstructure. In the building direction, there appears to be no gap, and a uniform thickness of 200 µm is observed. The average filament width is approximately 500 µm, resulting in a filament flattening ratio of about 250%. A closer inspection of the material shows small agglomerates of iron oxide particles (Figure 5b,c), along with evidence of particle pull-out and tearing. These observations suggest a quasi-ductile fracture behavior. In addition, the cracking pattern shows a cohesive laying with no particular inter-filament cracking in the building direction (Figure 5c).

In Figure 5d, the SEM micrograph reveals the interfacial debonding between the PLA matrix and the iron oxide particles. Some particles are partially embedded in the matrix, with visible gaps around them. These voids indicate a weak interface, limiting the load transfer from the matrix to the reinforcement (Figure 5d). This poor adhesion can lead to early crack propagation from the filaments in the external frame, as seen in Figure 4b.

**Figure 6:**
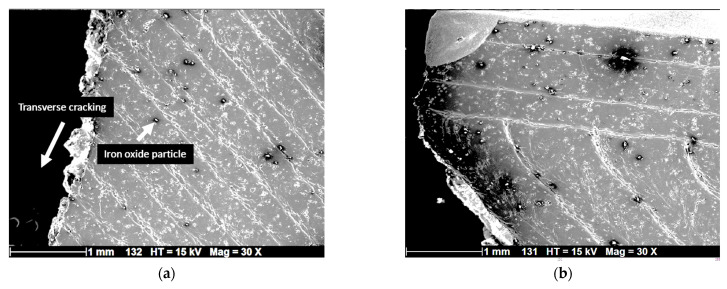
SEM micrographs showing top view of the cracking behavior, (**a**) top view showing cracking along the sample width; (**b**) a close view of ruptured filament close to the external frame.

Figure 6 shows SEM micrographs of ruptured specimens from the top view, displaying a relatively smooth surface texture within the plane of construction, with minor imperfections related to the presence of iron oxide particles on the filament surface. The fracture mechanism involves less tearing of the filaments, confirming that interfacial decohesion plays a critical role in triggering specimen rupture. Additionally, the limited inclination of crack propagation paths suggests a predominant opening mode, with no evident micro voids. The fracture surfaces display sharp, clean fracture lines, and no distinct microcracks are visible around the iron oxide particles in the PLA matrix (Figure 6b).

### 5.3. Microstructural Interpretation

**Figure 7:**
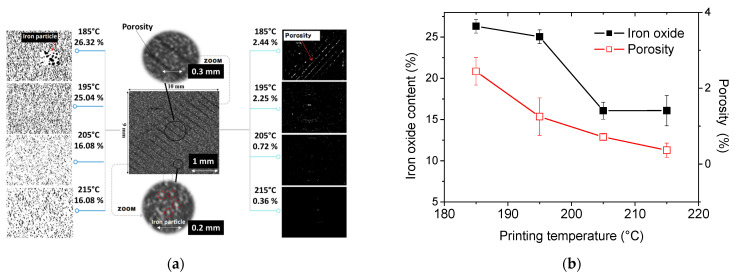
Result of X-ray microtomography image segmentation, (**a**) iron oxide particles and porosity in 3D-printed PLA–iron oxide composites as a function of printing temperature; (**b**) porosity and iron oxide volume contents as a function of the printing temperature.

Figure 7a presents the segmented X-ray microtomography images, showing the distribution of iron oxide particles and porosity in 3D-printed PLA-iron oxide composites at different printing temperatures. The iron phase is depicted in black, while porosity is shown in white.

The greyscale image in Figure 7a reveals the in-plane microstructure of the 3D-printed dog-bone sample, displaying the −45°/+45° layup pattern. Dark grey areas correspond to the low-density phase, representing porosity, while the iron oxide particles are visible in a lighter grey due to their higher density. On the right side, the segmented cross-section reveals the alignment of porosity following the same direction as the filament orientation. It is observed that porosity decreases as the printing temperature increases. On the left side, the segmented images of iron particles indicate a more uniform distribution of iron oxides, with no significant clustering.

To further quantify the phase properties, Figure 7b shows the porosity and iron oxide volume fractions as a function of printing temperature. Error bars are included to represent the variability observed across four different tomograms.

Figure 7b demonstrates that the percentage of iron oxide particles remains fairly consistent (25–26%) at lower printing temperatures but decreases to approximately 16% at higher temperatures. Considering the statistical reliability of the data, the apparent reduction in iron oxide content can be attributed to several factors. First, the resolution limit of the tomography prevents the detection of particles smaller than the voxel size, unless they form small clusters during printing. Second, differences in material flowability, rather than data variability, likely play a role. Indeed, the initial iron oxide content in the raw material is fixed and should not vary with printing temperature. Figure 7b therefore represents an apparent volume fraction of iron oxide in the printed composite, which may be influenced by material flow behavior, porosity, densification, and heterogeneity in particle distribution, rather than reflecting a real reduction in content. Furthermore, the flowability of iron oxide particles can be affected by the nozzle geometry, potentially causing local variations in particle concentration. At higher temperatures, this effect may be amplified, as partial settling of the particles within the nozzle could also contribute to the observed differences.

Assuming that PLA and iron oxide are incompressible materials, their respective volumes should remain constant within the same filaments, implying that only porosity could vary. In this case, a reduction in porosity should lead to an increase in the volume fraction of iron oxide. However, this is contrary to what we observed. If we assume that the iron oxide particles are on the nanometric scale and that SEM observations reveal agglomerations of these particles, it is possible that inter-particle porosity increases at low temperatures, where viscosity is high. Conversely, as the temperature rises, viscosity decreases, reducing the volume fraction of the iron oxide particle agglomerations. This hypothesis could be supported by further investigation using data from X-ray microtomography.

Regarding mechanical properties, the results of the connectivity index could explain the observations. The higher the temperature, the higher the connectivity index, which facilitates the rapid propagation of microcracks and leads to premature failure. Conversely, at low temperatures, higher inter-particle porosity could offset the effect of connectivity.

Figure 7b shows that porosity decreases as the printing temperature increases, dropping from 2.44% at 185 °C to just 0.36% at 215 °C. This reduction indicates improved cohesion between the printed layers, with fewer voids forming between adjacent filaments. Higher printing temperatures enhance layer fusion. Based on the results summarized in Table 1, the stability of the tensile performance across different printing temperatures can be attributed to the opposing effects of reduced porosity and reduced iron oxide content. While lower porosity tends to improve tensile performance, the decrease in iron oxide limits the reinforcing effect on the PLA matrix.

**Figure 8:**
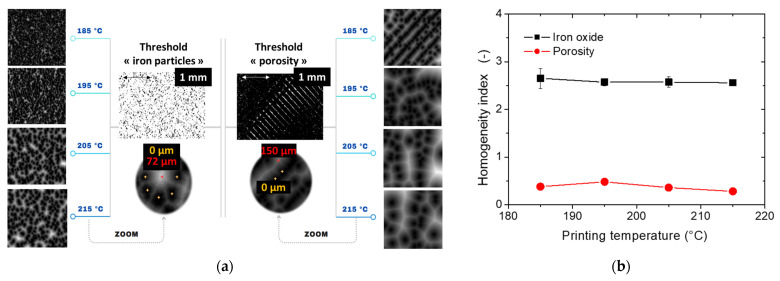
Image processing results. (**a**) Analysis of the spatial homogeneity of phases in 3D-printed PLA-iron oxide composites using Euclidian Distance Maps (EDM). (**b**) Homogeneity index of iron particles and porosities in 3D-printed PLA-iron oxide composites as a function of the printing temperature.

Figure 8a presents the spatial heterogeneity analysis of both iron oxide and porosity phases using the Euclidean Distance Map (EDM) technique. Specific patterns emerge particularly for a printing temperature of 185 °C, corresponding to the regular spatial arrangement of the porosities. In the construction plane, a dominant orientation of −45° is evident, aligning with the material extrusion direction, which reflects a printing angle of 0° (i.e., layups of −45°/+45°).

In contrast, the EDM distribution for iron particles exhibits a more random pattern, with no clear preferred orientation. Figure 8b provides a better quantification of homogeneity, using the homogeneity index derived from the slope of the EDM’s cumulative histogram. The homogeneity index for the iron oxide phase is higher than that of porosity, due to the preferred orientation of the porosity phase, which introduces a broad range of grey levels in the EDM image. The homogeneity index remains relatively stable across different printing temperatures.

The regular spatial distribution of iron particles also accounts for the cracking patterns observed in tensile tests (Figure 3). In a typical 3D-printed material with a printing angle of 0°, cracks are expected to propagate at either −45° or +45° due to porosity alignment in those directions. However, the dominant mode of crack opening appears to be driven by interfacial decohesion, which initiates randomly at the scale of the filament and then propagates in a direction perpendicular to the loading axis.

With regard to the overall trend of the spatial homogeneity of the phases with respect to the printing temperature, only a slight decrease in the homogeneity index is depicted, which can be related to an incremental improvement in the homogeneity.

**Figure 9:**
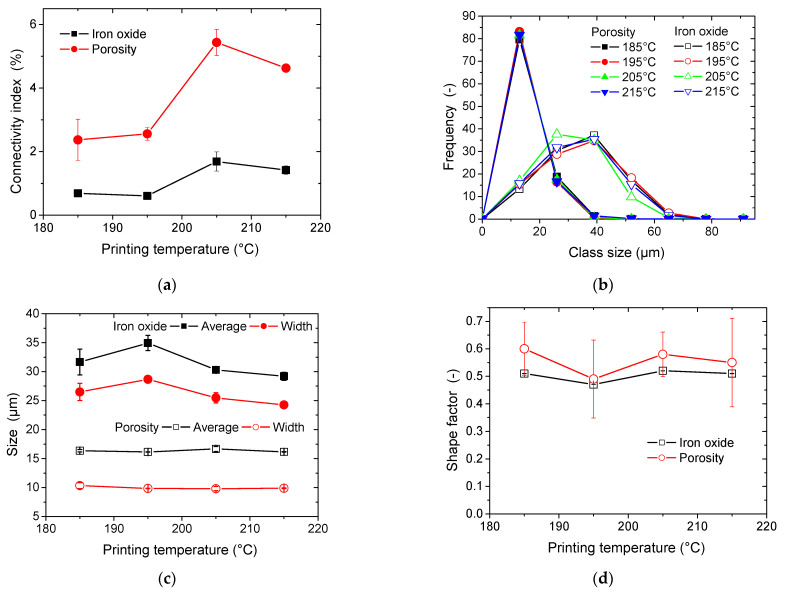
Quantitative image analysis results for 3D-printed PLA-iron oxide composites showing (**a**) phase connectivity index, (**b**) size distribution, (**c**) average feature size and width, and (**d**) shape factor for both iron oxide and porosity phases as a function of the printing temperature.

Figure 9a illustrates the result of 3D labelling for both iron oxide and porosity. The labelling is based on six-neighbour connectivity, allowing features sharing a surface to be part of the same entity. The iron oxide particle connectivity is lower compared to the porosity. It slightly increases with the rise in printing temperature. The amount of increase is limited, and for all cases a connectivity below 2% is depicted. This suggests that the iron particles tend to form clusters.

The connectivity rate for porosities follow the same trend with respect to the printing temperature, with the exception that rates as high as 5% are depicted. Although this is not a confirmed tendency, the relatively low connectivity ratio at 215 °C compared to the other printing temperatures may suggest that the low pore connectivity is correlated to the reduction in the porosity level in the 3D-printed composite. Figure 9b illustrates the evolution of the particle size distribution for the iron oxide phase. All distributions exhibit a similar symmetrical shape, capturing features as small as the voxel size. This symmetry allows for the entire size dispersion of iron oxides to be reasonably fitted with a Gaussian profile, without significant discrepancy. A slight shift towards smaller particle sizes is observed as the printing temperature increases, likely due to the breakage of particle clusters during the material extrusion process. The presence of these finer particles in the composite explains why high particle connectivity is maintained (Figure 9) at elevated temperatures, despite the reduction in particle content (Figure 7).

Using Gaussian curve fitting, the mean and width of the particle size distributions were determined and are presented in Figure 9c as a function of printing temperature. The relatively stable average particle size observed at 205 °C results from a balance between a pronounced shift toward smaller particle sizes (Figure 9b) and a reduced presence of larger size classes.

Figure 9c also presents the size distribution analysis for porosity in 3D-printed PLA-iron oxide composites. Most of the porosity profiles overlap, with notable changes only occurring in the larger pore size classes, where a slight shift toward larger pore sizes is observed at higher printing temperatures. Although this change is small, it seems to contradict the expectation of reduced porosity as printing temperature increases. The argument for better bonding and coalescence between adjacent filaments at higher temperatures remains valid. However, the increased proportion of iron particles, particularly finer ones at higher temperatures, contributes to the increase in porosity size. Notably, the larger porosity sizes are comparable to the average size of iron oxide particles (around 30 µm).

Figure 9d presents the morphology of both porosity and iron oxide phases using the shape factor descriptor. This descriptor quantifies the ratio between the largest and smallest dimensions of labelled features in each phase. Despite a high standard deviation in the morphology analysis, both phases show an elongated shape, as their shape factors are less than 1, which corresponds to a perfect circular shape. No clear trend related to printing temperature can be discerned from the average data. However, the shape factor for iron oxides appears to be lower, suggesting that the direction in which the filament is laid may affect particle clustering.

**Figure 10:**
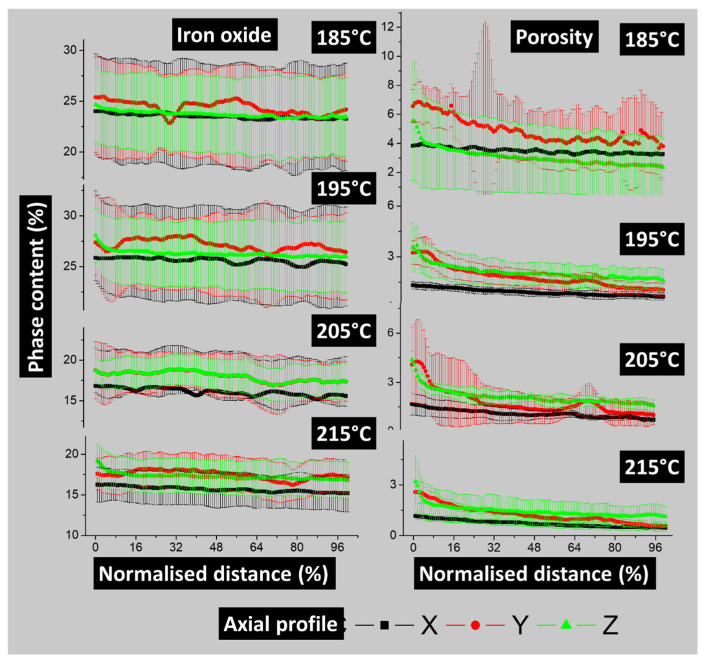
Axial phase content profiles in main directions of porosity and iron oxide phases as a function of the printing temperature.

The anisotropy of the phase spatial distribution is illustrated in Figure 10.

While there is a correlation between the heterogeneity index and how the phases are distributed across the three primary spatial directions, this index does not quantify the relative change in one direction compared to the others. Figure 10 offers a more detailed quantitative analysis of phase distribution anisotropy through axial porosity profiles. It presents the results for both porosity and iron oxide content in the X, Y, and Z directions.

The axial profiles of iron oxide content along the sample’s main directions show no significant anisotropy, as the profiles in all directions overlap. Additionally, the standard deviation measured in each plane from the 3D images reveals no discernible trend, except that the average iron oxide content is higher at lower printing temperatures, as also shown in Figure 7b. The trends for the porosity axial profiles are different. A separation between the X, Y, and Z profiles is more effective even if the standard deviation shows some overlapping.

### 5.4. Finite Element Simulation Results

The analysis of the experimental results highlights opposing factors that influence the tensile response of 3D-printed PLA-iron oxide composites. These opposing factors are the reduction of both the iron oxide content and porosity. In the case of a full load transfer across the interface between iron oxide and PLA, a larger content of iron oxides is supposed to enhance the performance due to its superior intrinsic properties compared to PLA, while porosity negatively impacts tensile performance. To better isolate and understand the individual contributions of these factors, finite element simulations are employed, using data obtained from X-ray microtomography.

**Figure 11:**
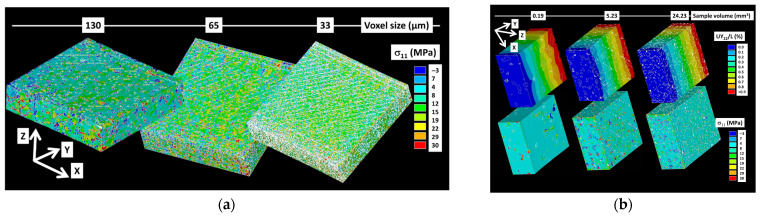
Finite element results. (**a**) Effect of resolution, and (**b**) sample size on the stress component σ_11_ distribution.

To ensure the predictions remain relevant with regard to resolution, the impact of increasing voxel size is explored in Figure 11a.

Calculations were carried out on the entire acquired image with voxel sizes increasing from 33 µm up to 130 µm. Under two-phase model conditions, computational times ranged from 60 to 2400 s per load increment. Stress counterplots reveal that key microstructural details, such as filament orientation and the external frame, are captured effectively for voxel sizes up to 65 µm. At finer scales, the observed stress heterogeneity stems from the presence of iron oxides and process-induced porosity. On a microstructural level, stress heterogeneity also reflects the influence of filament arrangement, particularly at a voxel size of 33 µm. Notably, no layering effect is observed in the stress variation along the building direction, confirming the cohesive layering seen in the SEM micrographs (Figure 5c).

Figure 11b presents the effect of sampling on the predicted stress component $\sigma_{11}$ distribution. These computations were performed on as-acquired volumes with the original voxel size of 6.5 µm. Figure 11b also displays the displacement field under X-direction loading, representing specimen length. In a sample volume of 190 × 10^6^ µm^3^, stress distribution varies notably based on whether a porosity feature is present. Fewer microstructural details and stress heterogeneities are captured, with stress concentrations forming around porosity as well as isolated areas with iron oxides. In larger sample volumes over 5 mm^3^, the alignment of porosity tends to influence stress distribution; however, limited porosity connectivity restricts high stress concentrations. As such, alternating high and low-stress fields are not observed in sampled regions, though stress heterogeneity remains due to iron oxide presence.

**Figure 12:**
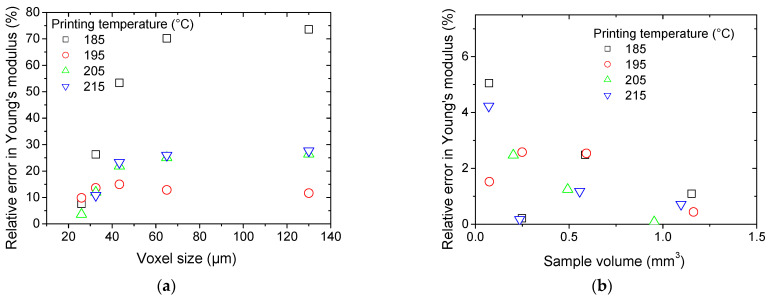
Finite element results showing the effect of (**a**) resolution, and (**b**) sampling on the variation in predicted Young’s modulus.

The relative error in Young’s moduli with respect to the value computed for a voxel size of 20 µm is plotted against the voxel size in Figure 12a.

A high dependence of Young’s modulus on the resolution is predicted for samples printed using a printing temperature of 185 °C. The relative error in Young’s modulus evaluation falls within 10% for voxel sizes below 30 µm.

For the two-phase model, the predicted Young’s modulus in the X-direction is plotted against the sample volume in Figure 12b for all samples. The voxel size is kept at its original value of 6.5 µm. The reference region of interest represents 30% of the entire acquired volume, which corresponds to models as large as 86 × 10^6^ dof. A stable trend is obtained for all samples where the relative error in Young’s modulus is found to be below 10% for ROI volumes as small as 0.03 mm^3^. This remarkable stability can be related to the spatial homogeneity of iron oxide particles rather than to porosity.

**Figure 13:**
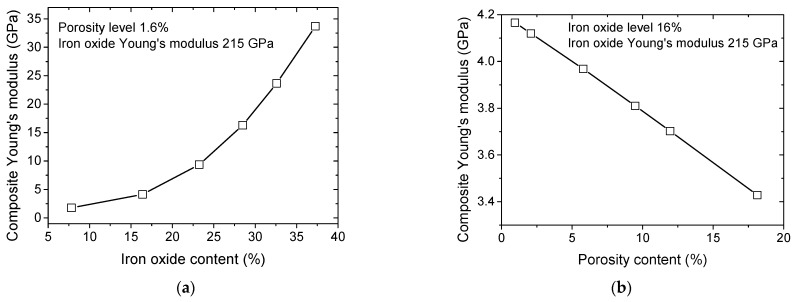
Effect of iron oxide content and porosity on the predicted Young’s modulus of PLA–iron oxide composites using a two-phase model. (**a**) Young’s modulus as a function of iron oxide content; (**b**) Young’s modulus as a function of porosity level.

In order to discriminate the role of porous structure and the iron oxide phase, simulations are considered by varying phase contents sequentially based on a two-phase model, as shown in Figure 13.

These simulations are performed assuming an increasing phase content by artificially shifting the grey level threshold to control the iron oxide and PLA contents while maintaining the main microstructural features. The predicted Young’s modulus of the composite is plotted against phase content in Figure 13. The effect of iron oxide content on the predicted stiffness of the 3D-printed composite is depicted in Figure 13a for a fixed porosity content of 1.6%. This porosity content is selected to be in the middle range of the porosity contents observed for all 3D-printed samples (Figure 7b). The predicted composite Young’s modulus exhibits an exponential trend with respect to the iron oxide content due to the large contrast between PLA and iron oxide stiffness. Within the experimentally observed range of iron oxide contents, determined by X-ray microtomography (Figure 7b), the predicted Young’s modulus is notably higher than the values listed in Table 1. This suggests that load transfer across the PLA-iron oxide interface is modified, as indicated by SEM observations (Figure 5).

The predicted trend can be approximated using an exponential growth function of the form

(5) $$E_{P/I}\left(GPa\right)=457+644\times exp\left(f_{I}\left(\%\right)/10\right);\;R^{2}=0.997$$

where $E_{P/I}$ is the predicted composite Young’s modulus, and $f_{I}$ is the iron oxide content.

In order for the predicted value to match the experimental one, the iron content needs to be of the order of 1%.

Additionally, for a fixed iron oxide content of 20%, increasing the porosity level from 1% to 10% results in only a slight decrease in the composite Young’s modulus (Figure 13b). The overall stiffness of the composite at this filler content is dominated by the matrix–particle interaction and the arrangement of iron oxide particles, rather than by isolated voids. This linear decrease is much lower than the nonlinear increase of Young’s modulus driven by the change in iron oxide content. This indicates that porosity content has a negligible effect compared to iron oxide content for all printing conditions. A linear approximation of the trend between composite Young’s modulus and porosity level provides the following correlation,

(6) $$E_{P/I}\left(GPa\right)=5545-69\times f_{P}\left(\%\right);\;R^{2}=1.00$$

where $f_{P}$ is the porosity level.

The porosity level required during processing to match the experimental data is approximately 65%, which is an order of magnitude higher than the observed level.

**Figure 14:**
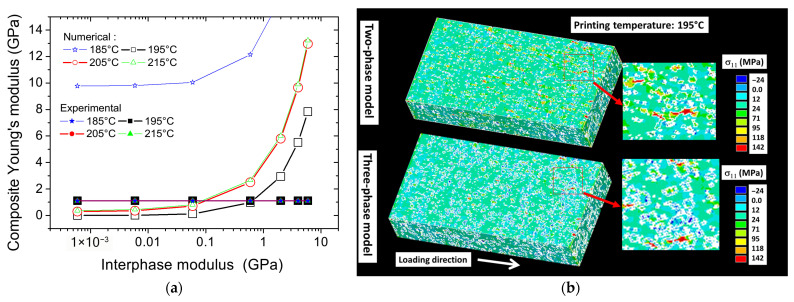
Finite element results: (**a**) identification of interphase Young’s modulus for all considered printing temperatures according to three-phase model implementation; (**b**) illustrations of stress component σ_11_ counterplots for both two-phase and three-phase models.

To account for the local variation in stiffness across the interface, a three-phase model is applied. Figure 14a presents the predicted composite Young’s modulus as a function of the interphase modulus, assuming a fixed interphase thickness of 33 µm. The graph also includes the experimental Young’s modulus of the PLA-iron oxide composite for all printing temperatures. A positive nonlinear correlation is found between the interphase modulus and the overall composite modulus. The interphase modulus represents from these simulations a leading effect similarly to the iron oxide effect. Indeed, an exponential form is predicted for all printing temperatures where predicted composite moduli below 1 GPa are obtained for interphase moduli of the order of 100 MPa. The best matching between the numerical results and the experimental evidence is found for an interphase modulus in the range 687 and 142 MPa, which is lower than Young’s modulus of PLA. This is the case for most conditions except for the printing temperature of 185 °C. This discrepancy can be explained by a weaker interface condition, triggering a larger interphase thickness, or an underestimation of the exact interface quantity. Despite this outlier, the study demonstrates that the three-phase model offers a significantly improved representation of the mechanical behavior compared to the two-phase model, which fails to adequately account for the observed stiffness reduction (Figure 14a). By explicitly incorporating the interphase, the three-phase model captures its contribution more accurately, estimating the interphase stiffness to be between 13% and 63% of the PLA matrix stiffness.

Figure 14b shows the predicted stress counterplots for both two-phase and three-phase models, where the printing temperature is fixed to 195 °C and the interphase modulus is fixed to 687 MPa. Significant changes in the stress distributions are observed between two-phase and three-phase models. Firstly, lower stress levels are triggered by the presence of an additional contrast in material stiffness when using the three-phase model. In addition, for the same three-phase model, the presence of larger areas under compressive stress is observed due to Poisson’s contraction. And, finally, stress heterogeneity is also further intensified by the abrupt change in stiffness across the interfaces.

## 6. Magnetic Actuation of 3D-Printed PLA-Iron Oxide

**Figure 15:**
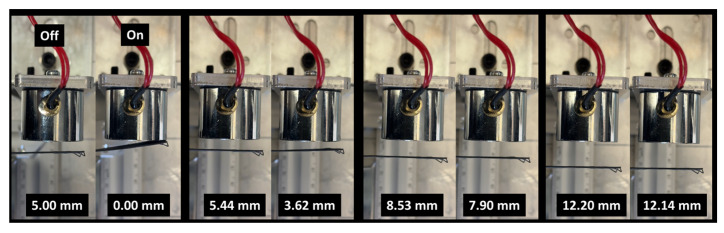
Magnetic actuation of a 3D-printed PLA–iron oxide strip of 0.4 mm in thickness under an external electromagnet. Images show the strip in the field-off and field-on states at different initial displacements, demonstrating reversible bending response toward the magnet.

To gain deeper insight into the actuation performance of the PLA-iron oxide composite, the bending behavior of a strip with a cross-sectional area of 83 mm × 22 mm is examined. The effective gauge length is 53 mm. Figure 15 illustrates the lateral displacement of a 3D-printed strip with a thickness of 0.4 mm under the influence of an applied magnetic field.

**Figure 16:**
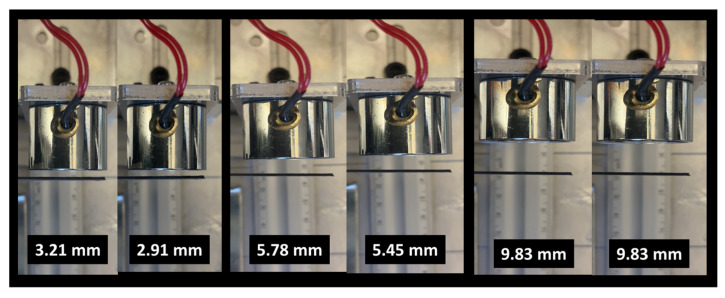
Magnetic actuation of a 3D-printed PLA-iron oxide strip with a thickness of 0.6 mm under an external electromagnet. Images show the strip in the field-off and field-on states at different initial displacements, demonstrating a reduced bending response compared to the thinner 0.4 mm strip.

The left column of each pair corresponds to the field-off state, while the right column shows the field-on state. In the absence of the magnetic field, the strip remains nearly straight, with minimal displacement values recorded (e.g., 5.00 mm, 5.44 mm, 8.53 mm, and 12.20 mm across different positions). When the magnetic field is activated, the embedded iron oxide particles interact with the field, inducing a clear bending deformation. This is reflected by the reduced displacement values (0.00 mm, 3.62 mm, 7.90 mm, and 12.14 mm, respectively), indicating a lateral movement of the strip towards the magnet. The progressive increase in displacement with increasing initial distance suggests a field-dependent actuation capability, where the extent of bending is influenced by both the magnetic field strength and the geometry of the strip. A comparison between the 0.4 mm- and 0.6 mm-thick PLA–-ron oxide strips highlights the strong influence of specimen thickness on magnetic actuation. Indeed, the thinner strip (0.4 mm) exhibited larger bending displacements when exposed to the magnetic field, with clear differences observed between the field-off and field-on states. In contrast, the thicker strip (0.6 mm) showed only marginal changes in displacement under identical conditions (Figure 16), with the final measured positions remaining nearly unchanged.

This reduction in actuation efficiency can be attributed to the higher bending stiffness of the thicker plate, which scales with the cube of thickness, thereby significantly limiting the deformation induced by the magnetic force.

**Figure 17:**
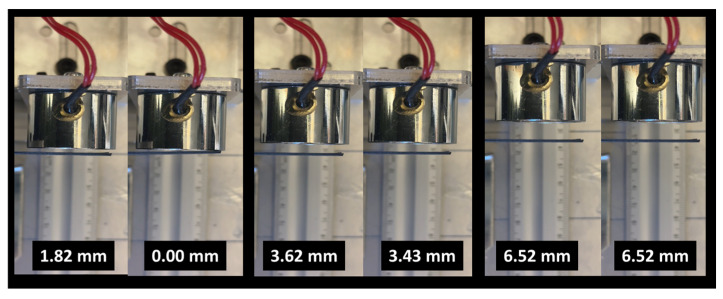
Magnetic actuation of a 3D-printed PLA-iron oxide strip with a thickness of 0.8 mm under an external electromagnet. Images show the strip in the field-off and field-on states at different initial displacements, demonstrating a reduced bending response compared to the thinner 0.4 mm strip.

The magnetic actuation of the 0.8 mm thick 3D-printed PLA-iron oxide plate under an external electromagnet shows a significantly reduced bending response compared to thinner samples (Figure 17). As seen from the field-off and field-on states at different initial displacements, the change in deflection is minimal, indicating that the increased stiffness of the thicker plate strongly limits its ability to deform under magnetic torque. Even at larger initial displacements, the magnetic effect is barely distinguishable, demonstrating that at this thickness the elastic resistance of the material dominates over the applied magnetic force, thereby reducing actuation efficiency and highlighting the critical role of thickness optimization in achieving effective magnetic responsiveness.

**Figure 18:**
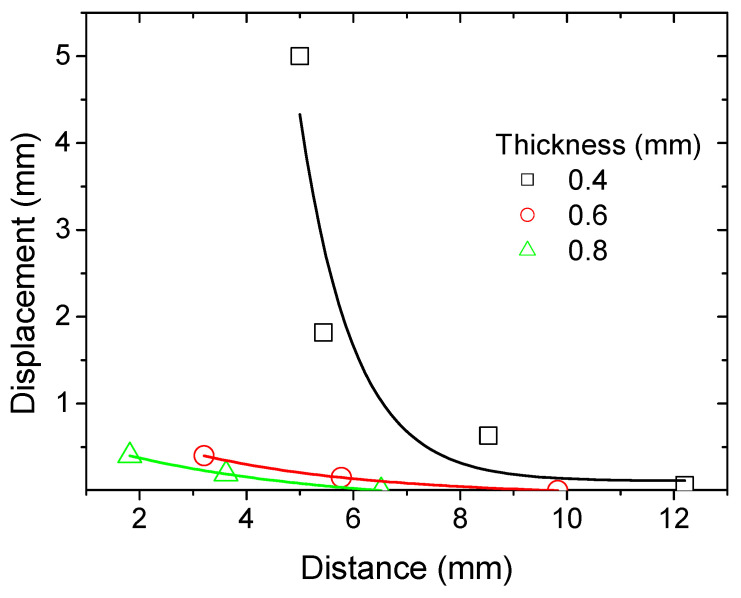
Quantification of the magnetic actuation of a 3D-printed PLA-iron oxide strip as a function of the thickness under an external electromagnet.

Figure 18 shows the plot of the induced displacement by magnetic actuation versus distance between the strip and the electromagnet of varying thickness. A general decrease in displacement is observed with increasing distance for all samples. The magnitude of displacement at a given distance is inversely related to plate thickness, indicating that structural stiffness is a key factor in magnetic actuation performance. The observed displacement exhibits a clear inverse relationship with distance for all plate thicknesses, consistent with the expected decay of magnetic force strength. Crucially, plate thickness significantly modulates this response: the thinnest plate (0.4 mm) demonstrates the largest displacement, followed by the intermediate (0.6 mm), with the thickest plate (0.8 mm) showing the smallest. This trend indicates that magnetic actuation is more effective on thinner plates, as their lower structural stiffness offers less resistance to deformation from the applied magnetic force. While this thickness-dependent effect is most pronounced at shorter distances where the magnetic force is strongest, the displacement for all plates converges toward zero at larger distances, confirming that the influence of distance ultimately dominates over that of plate thickness. In conclusion, increased plate thickness reduces magnetic actuation displacement, with thinner plates proving to be more responsive and thicker plates more resistant to deformation.

## 7. Conclusions

This study demonstrates that varying the printing temperature between 185 °C and 215 °C during the 3D printing of PLA reinforced with iron oxide particles results in modest changes to tensile behavior. SEM analysis identifies interfacial debonding mechanisms as the primary factor limiting effective load transfer.

Further investigation using X-ray microtomography concludes on a reduction in both porosity and iron oxide contents with increasing printing temperature, typically ranging from (26%, 16%) for porosity and (2.4%, 0.4%) for iron oxide. These trends are attributed to improved inter-filament adhesion and differences in material flow behavior.

Finite element analysis highlights the key factors affecting the tensile behavior and stiffness of 3D-printed PLA-iron oxide composites. The two-phase model reveals that the inclusion of iron oxide significantly enhances stiffness due to its superior mechanical properties, while porosity has a negligible impact.

The closest agreement between experimental results and numerical predictions is achieved with a three-phase model. This model accounts for the varying phase content, the lack of effective load transfer between the matrix and reinforcement, and the relatively minor changes in tensile performance under different printing conditions. The three-phase model estimates the interphase modulus to range between 687 MPa and 142 MPa, which aligns with most printing conditions, except for a printing temperature of 185 °C.

The magnetic actuation experiments on the 0.8 mm PLA-iron oxide plate demonstrate a strongly thickness-limited response, with the largest observed deflection reduction being 1.82 mm, while intermediate and large initial displacements yield only 0.19 mm (~5.3%) and 0.00 mm (0%), respectively. On average, the absolute actuation is limited to ≈0.67 mm, confirming that the increased stiffness of the thicker plate suppresses magnetic responsiveness, particularly at higher displacements where elastic resistance dominates the applied torque.

Looking ahead, further exploration of the interphase’s local behavior and the influence of more impactful printing parameters could enable better optimization of the composite’s performance, particularly in relation to magnetic actuation applications. As a future direction, the data presented in this study will serve as a foundation for a broader research effort focused on coupling magnetic actuation with mechanical testing, including flexural bending. This will involve incorporating multi-physics modelling and examining the effects of further process parameters such as layer height, infill density, and post-processing treatments on magnetic response. These investigations are currently in progress.

## Figures and Tables

**Table 1:** The mechanical characteristics of a 3D-printed PLA-iron oxide composite in relation to the printing temperature. E_Y_: Young’s modulus, σ_Y_: yield stress, σ_M_: tensile strength, σ_R_: ultimate stress, ε_R_: elongation at break.

Tp (°C)	E_Y_ (MPa)	σ_Y_ (MPa)	σ_M_ (MPa)	σ_R_ (MPa)	ε_R_ (%)
185	1112 ± 5	39.3 ± 0.7	45.9 ± 0.8	41.2 ± 1.0	5.4 ± 0.3
195	1102 ± 23	38.7 ± 0.6	45.1 ± 0.9	42.6 ± 1.8	5.3 ± 0.4
205	1108 ± 13	38.6 ± 0.5	45.0 ± 0.8	41.8 ± 1.8	5.3 ± 0.2
215	1096 ± 16	37.9 ± 0.4	44.1 ± 0.4	41.3 ± 0.9	5.2 ± 0.3

## Data Availability

Data are contained within the article.

## Supplementary Materials

The following supporting information can be downloaded at: https://www.mdpi.com/article/10.3390/polym17182485/s1, Figure S1: Literature analysis of additive manufacturing publications (August 2025): (a) Overview of publications retrieved using the keyword “additive manufacturing” (3935 records in total). Filtering for studies involving metal-based feedstock materials yields 846 publications (~22%). (b) Refined analysis focusing specifically on metal-based feedstock materials. (c) Further filtering for magnetic components within metal-based feedstock materials reveals only seven relevant publications (<1%), with no results found when using the keyword “magnetite”.
