# Supplementary material for: Effect of Printing Temperature on the Microstructure and Tensile Properties of Polylactic Acid–Magnetic Iron Composites Manufactured by Material Extrusion
Source: Polymers (Basel). 2025 Sep 14;17(18):2485. doi: 10.3390/polym17182485 (PMC12473559; doi:10.3390/polym17182485)
Supplement: Supplementary file 1 [file polymers-17-02485-s001.zip › polymers-3742461-supplementary.pdf]

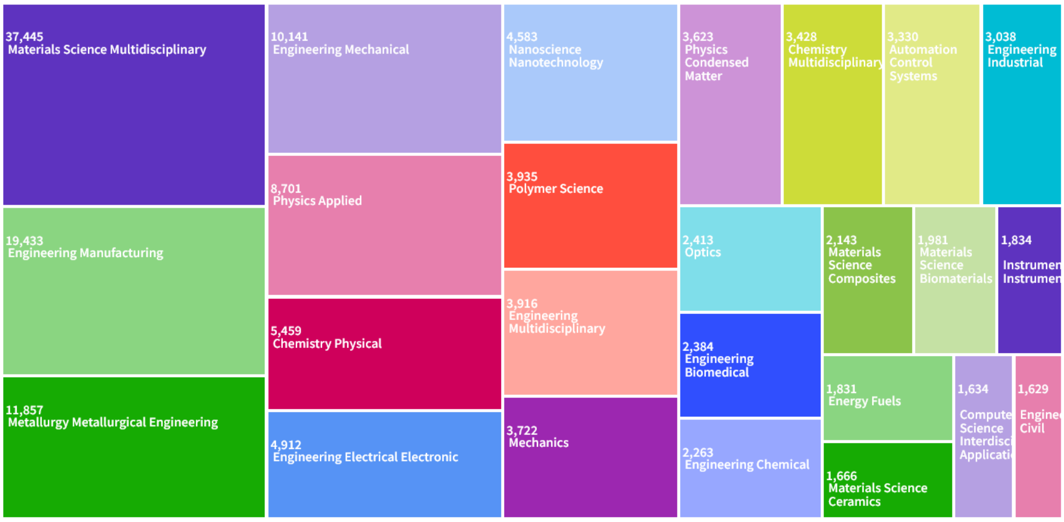
37,445
Materials Science Multidisciplinary
10,141
Engineering Mechanical
4,583
Nanoscience Nanotechnology
3,623
Physics Condensed Matter
3,428
Chemistry Multidisciplinary
3,330
Automation Control Systems
3,038
Engineering Industrial
8,701
Physics Applied
3,935
Polymer Science
19,433
Engineering Manufacturing
2,413
Optics
2,143
Materials Science Composites
1,981
Materials Science Biomaterials
1,834
Instrumen
Instrumen
3,916
Engineering Multidisciplinary
5,459
Chemistry Physical
2,384
Engineering Biomedical
1,831
Energy Fuels
1,634
Compute
Science
Interdisc
Applicati
1,629
Engine
Civil
11,857
Metallurgy Metallurgical Engineering
3,722
Mechanics
4,912
Engineering Electrical Electronic
2,263
Engineering Chemical
1,666
Materials Science Ceramics

(a)

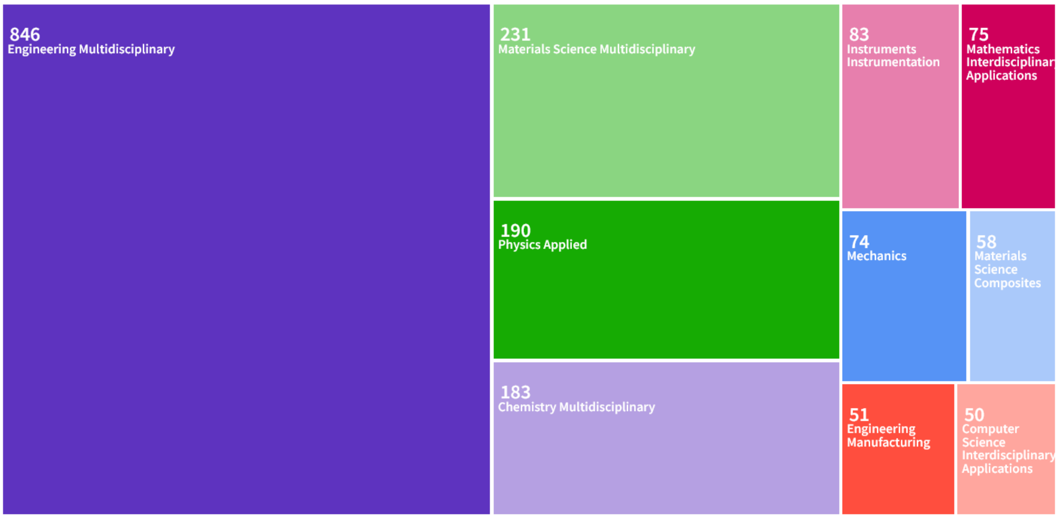
846
Engineering Multidisciplinary
231
Materials Science Multidisciplinary
83
Instruments Instrumentation
75
Mathematics Interdisciplinar Applications
190
Physics Applied
74
Mechanics
58
Materials Science Composites
183
Chemistry Multidisciplinary
51
Engineering Manufacturing
50
Computer Science Interdisciplinary Applications

(b)

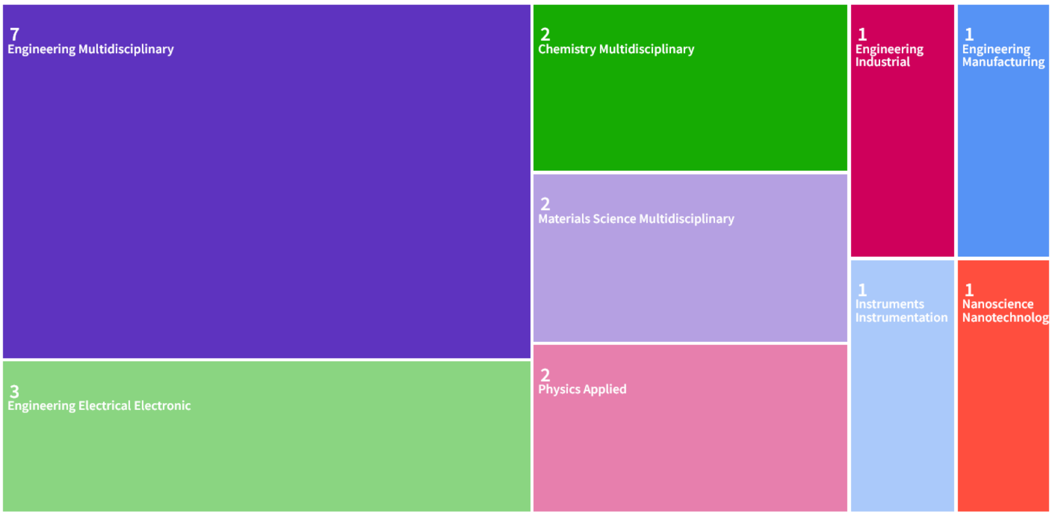
7
Engineering Multidisciplinary
2
Chemistry Multidisciplinary
1
Engineering Industrial
1
Engineering Manufacturing
2
Materials Science Multidisciplinary
1
Instruments Instrumentation
1
Nanoscience Nanotechnolog
3
Engineering Electrical Electronic
2
Physics Applied

(c)

Figure S1. Literature analysis of additive manufacturing publications (August 2025): (a) Overview of publications retrieved using the keyword "additive manufacturing" (3,935 records in total). Filtering for studies involving metal-based feedstock materials yields 846 publications (~22%). (b) Refined analysis focusing specifically on metal-based feedstock materials. (c) Further filtering for magnetic components within metal-based feedstock materials reveals only seven relevant publications (<1%), with no results found when using the keyword "magnetite".
